# Association between chymase gene polymorphisms and atrial fibrillation in Chinese Han population

**DOI:** 10.1186/s12872-019-01300-7

**Published:** 2019-12-30

**Authors:** Dongchen Zhou, Yuewei Chen, Jiaxin Wu, Jiabo Shen, Yushan Shang, Liangrong Zheng, Xudong Xie

**Affiliations:** Department of Cardiology, First Affiliated Hospital, School of Medicine, Zhejiang University, Hangzhou, China

**Keywords:** Atrial fibrillation, *CMA1*, Chymase, Single nucleotide polymorphism, Angiotensin II

## Abstract

**Background:**

Chymase is the major angiotensin II (Ang II)-forming enzyme in cardiovascular tissue, with an important role in atrial remodeling. This study aimed to examine the association between chymase 1 gene (*CMA1*) polymorphisms and atrial fibrillation (AF) in a Chinese Han population.

**Methods:**

This case-control study enrolled 126 patients with lone AF and 120 age- and sex-matched healthy controls, all from a Chinese Han population. Five *CMA1* polymorphisms were genotyped.

**Results:**

The *CMA1* polymorphism rs1800875 (G-1903A) was associated with AF. The frequency of the GG genotype was significantly higher in AF patients compared with controls (*p* = 0.009). Haplotype analysis further demonstrated an increased risk of AF associated with the rs1800875-G haplotype (Hap8 TGTTG, odds ratio (OR) = 1.668, 95% CI 1.132–2.458, *p* = 0.009), and a decreased risk for the rs1800875-A haplotype (Hap5 TATTG, OR = 0.178, 95% CI 0.042–0.749, *p* = 0.008).

**Conclusions:**

*CMA1* polymorphisms may be associated with AF, and the rs1800875 GG genotype might be a susceptibility factor for AF in the Chinese Han population.

## Background

Atrial fibrillation (AF) is the most common type of sustained clinical arrhythmia and is associated with significant cardiovascular morbidity and mortality. However, despite its clinical significance and high prevalence, the molecular basis of AF remains unclear. Diverse structural heart diseases and systemic disorders have been associated with AF, including valvular heart disease, ischemic heart disease, congestive heart failure, cardiomyopathy, pulmonary heart disease, cardiothoracic surgery, essential hypertension, and hyperthyroidism [1, 2], while age, obesity, obstructive sleep apnea–hypopnea syndrome, smoking, excessive drinking, and drug or toxicant use are also considered to contribute to the development of AF [1, 3]. However, AF outcomes differ among individuals with similar pathologies and comorbidities. Furthermore, up to a third of AF cases, termed ‘lone AF’, occur in the absence of identifiable underlying cardiovascular or other comorbid diseases [4]. The Framingham Heart Study showed that 30% of participants with AF had at least one parent with AF, and individuals with at least one parent suffering from AF had an approximately 40% increased risk of developing AF, after adjusting for age, sex, blood pressure, diabetes, and clinically overt heart disease [5]. These facts indicate a potential role for genetic variations in the pathogenesis of AF. Genome-wide association studies have identified common genetic variants associated with AF, and a recent report found three independent single nucleotide polymorphisms (SNPs) (rs2200733, rs17570669, and rs3853445) associated with increased AF risk [6]. Nevertheless, the genetic basis of AF pathogenesis is complex, involving modest contributions to disease risk from genetic variations in many genes, and further studies are needed to clarify the genetic determinants of AF risk.

The renin-angiotensin system (RAS) is involved in multiple pathophysiological processes in cardiovascular diseases and plays an important role in the development of AF [7]. Angiotensin II (Ang II) is the primary active component of RAS [8]. There is growing evidence to indicate that cardiac Ang II formation depends largely on the serine protease chymase [9], which is present in specific tissues, including the cardiac interstitium and blood vessels [10, 11], and converts angiotensin (1–12) to Ang II. Due to the difficulty in obtaining the samples of human heart tissue, most of the studies on chymase and Ang II are animal experiments. Most rodents, such as the mouse, rat, rabbit and pig, have β-chymase isoform in addition to α-chymase [12–14]. Humans only express the α-chymase. So the current experimental studies may not really represent the human condition. Chymase 1 (*CMA*1) gene is located on the long arm of chromosome 14 [15], and polymorphisms in *CMA1* have been reported to be associated with asthma, heart failure, and even aortic stenosis [15–17]. Many animal studies showed the benefits of chymase inhibitors to the cardiovascular system, such as preventing vascular proliferation [18], improving cardiac function [19] and inhibiting cardiovascular remodeling [20], etc., although studies in human body are still relatively rare. Chymase inhibitors have been shown to suppress cardiac remodeling and myocardial fibrosis [20], which are sufficient substrates for AF. However, the association between *CMA*1 polymorphisms and AF has not yet been reported. In this study, we investigated the association between *CMA*1 polymorphisms and AF with the aimed of identifying possible genetic variants of *CMA*1 that may predispose individuals to the risk of AF in the Chinese Han population.

## Methods

### Subjects

Lone AF was defined as AF without clinical or echocardiographic findings of cardiovascular diseases, hypertension, or metabolic or pulmonary diseases [21]. A total of 126 consecutive patients with lone AF admitted to the Department of Cardiology at the First Affiliated Hospital, School of Medicine, Zhejiang University, were recruited from January 2014 to December 2015. A group of 120 healthy age- and sex-matched individuals were enrolled as controls. The controls were recruited mainly for people with the habit of regular physical examination, so as to obtain more detailed and accurate history of past and present diseases. Both the case and control groups comprised Chinese Han individuals, all of whom had undergone clinical evaluation and echocardiographic examination. Patients’ medication included only agents to treat atrial fibrillation, such as amiodarone, digoxinum, or metoprolol, or anticoagulant drugs such as warfarin or rivaroxaban, and so on. All patients provided signed informed consent. The study conformed to the principles of the Declaration of Helsinki, and was approved by the Ethics Committee of the First Affiliated Hospital, School of Medicine, Zhejiang University (2016496). All serum samples and peripheral blood leukocytes were stored at − 80 °C for further measurements.

### Polymorphism identification and genotype analysis

We identified five SNPs (rs1800875, rs1800876, rs1885108, rs1956921 and rs5244) in the *CMA1* gene, according to previous studies [22]. Genomic DNA was extracted from peripheral blood leukocytes as described previously [23]. Five SNPs were genotyped in all 246 subjects using polymerase chain reaction (PCR) and direct sequencing. The primers, annealing temperature, length of PCR products, and the specific procedures have been described previously [22, 24]. The PCR products were subjected to PCR-direct sequencing using BigDye 3.1 chemistry and an ABI3500xl genetic analyzer (Applied Biosystems, Foster City, CA, USA), according to the manufacturer’s protocol [17]. The results were analyzed using DNAStar software SeqMan (DNAStar, Madison, WI, USA).

### Statistical analysis

Statistical analyses were performed using SPSS 22 software, with a value of *p* < 0.05 considered to be significant. The data were generally expressed as rates and ratios. Continuous variables were described as mean ± standard deviation. The Hardy–Weinberg equilibrium of each SNP was assessed by χ^2^ tests. Genotype and allele distributions were compared between AF patients and controls by χ^2^ tests. Odds ratios (ORs) were calculated using four-fold table χ^2^ analysis, and 95% confidence intervals (95% CI) and two-tailed *p* values were determined for each OR. Comparisons between groups were made using two-sample *t*-tests for continuous data. Only main haplotypes with frequencies > 1% were considered for further analysis. Possible associations between statistically inferred haplotypes and AF were calculated using SHEsis software (http://analysis.bio-x.cn/myAnalysis.php).

## Results

### Clinical characteristics

Table 1 shows the clinical characteristics of patients with controls and AF patients. As shown, patients with AF exhibited significantly lower total cholesterol (TC), and low density lipoprotein (LDL) compared to control subjects. There was no significant difference between the cases and controls for age, gender, BP, body mass index (BMI), triglyceride (TG) and glucose (Glu).

**Table 1:** Baseline characteristics of the study subjects

Parameters	controls	AF	*p*-value
Number	120	126	
Sex(male/female)	77/43	78/46	0.837
Age (years)	54.80 ± 12.99	55.80 ± 10.06	0.502
SBP (mm Hg)	124.62 ± 13.95	124.45 ± 15.32	0.931
DBP (mm Hg)	76.35 ± 8.71	76.18 ± 11.31	0.898
BMI (kg/m^2^)	23.50 ± 2.38	23.80 ± 3.30	0.530
TG (mmol/L)	1.17 ± 0.58	1.30 ± 0.60	0.094
TC (mmol/L)	4.46 ± 0.78	4.12 ± 0.86	0.002
LDL (mmol/L)	2.54 ± 0.60	2.33 ± 0.63	0.010
Glu (mmol/L)	4.68 ± 0.63	4.67 ± 0.63	0.829

*BMI* Body mass index, *SBP* Systolic blood pressure, *DBP* Diastolic blood pressure, *TC* Total cholesterol, *TG* Triglyceride, *HDL* High density lipoprotein, *LDL* Low density lipoprotein, *Glu* Glucose

### Genotype and allele frequencies

Five *CMA1* SNPs were genotyped in healthy controls and AF patients, and the distributions of most of the loci except the rs1800876 in AF patients were in Hardy–Weinberg equilibrium (*p* > 0.05) (Table 2). The genotype and allele frequencies of the five polymorphisms in AF patients are listed in Table 2. The minor allele frequencies of each SNP in the healthy Chinese Han population were as follows: rs1800875, 30.83%; rs1956921, 16.39%; rs5244, 25.86%; rs1800876, 37.82%; and rs1885108, 41.67% (Table 2).

**Table 2:** Genotype and allele distributions in Atrial fibrillation patients and controls

Variant	Allele	Group	n	Genotype (frequency)			*p*	OR (95% CI), *p*	Allele (frequency)		OR (95%CI), *p*	Hardy-Weinberg equilibrium	
	1/2			1/1	1/2	2/2		1/1 + 1/2vs 2/2	1	2	1 vs 2	χ2	*p*
rs1956921	C/T	AF	126	2 (1.59%)	25 (19.84%)	99 (78.57%)	0.285	0.629 (0.353–1.212) 0.114	29 (11.51%)	223 (88.49%)	0.664 (0.396–1.113) 0.118	0.084	0.7719
		controls	119	3 (2.52%)	33 (27.73%)	83 (69.75%)			39 (16.39%)	199 (83.61%)		0.017	0.8960
rs1800875	A/G	AF	124	10 (8.06%)	38 (30.65%)	76 (81.29%)	0.009	0.500 (0.300–0.832) 0.007	58 (23.39%)	190 (76.61%)	0.685 (0.458–1.023) 0.064	2.601	0.1068
		controls	120	7 (5.83%)	60 (50.00%)	53 (44.17%)			74 (30.83%)	166 (69.17%)		3.561	0.0592
rs1800876	T/C	AF	119	23 (19.33%)	59 (49.58%)	37 (31.09%)	0.082	1.122 (0.652–1.932) 0.678	105 (44.12%)	133 (55.88%)	1.298 (0.900–1.872) 0.162	5.502	0.0190
		controls	119	11 (9.24%)	68 (57.14%)	40 (33.62%)			90 (37.82%)	148 (62.18%)		0.036	0.9520
sc5244	C/T	AF	126	11 (8.73%)	44 (34.92%)	71 (56.35%)	0.225	0.859 (0.518–1.426) 0.557	66 (26.19%)	186 (73.81%)	1.017 (0.677–1.527) 0.934	1.180	0.2774
		controls	116	5 (4.31%)	50 (43.10%)	61 (52.59%)			60 (25.86%)	172 (74.14%)		1.785	0.1816
rs1885108	A/G	AF	126	16 (12.70%)	60 (47.62%)	50 (39.68%)	0.471	0.732 (0.434–1.234) 0.241	92 (36.51%)	160 (63.49%)	0.805 (0.560–1.157) 0.241	0.093	0.7603
		controls	120	19 (15.83%)	62 (51.67%)	39 (32.50%)			100 (41.67%)	140 (58.33%)		0.474	0.4911

*CI* Confidence interval, *OR* Odds ratio. The minor allele was referred to as allele 1 and the major allele as allele 2. OR estimated by two-tailed *P* values and 95% confidence intervals (95% CI)

The distribution of the rs1800875 (G-1903A) genotype differed significantly between AF patients and controls (*p* = 0.009). The observed genotype frequencies in AF patients and controls were 8.06 and 5.83% for AA, 30.65 and 50.00% for AG, and 81.29 and 44.17% for GG, respectively. The OR of the GG genotype for AF was 0.500 (95%CI, 0.300–0.832; *p* = 0.007). There were no significant differences in genotype or allele distributions for rs1800876, rs1885108, rs1956921, and rs5244 between the AF and control groups (*p* > 0.05).

### Haplotype analysis

The *CMA1* haplotypes were constructed according to their SNPs. Five *CMA1* haplotypes had frequencies > 1% in both AF patients and controls (Table 3). The occurrence of Hap8 TGTTG was significantly higher in AF patients than in controls (*p* = 0.009), and four-fold table χ^2^ analysis indicated that this haplotype might be associated with increased genetic susceptibility to AF (OR = 1.668, 95% CI 1.132–2.458). In contrast, Hap5 TATTG had a significantly lower incidence in AF patients compared with controls (*p* = 0.008), indicating a reduced risk of AF (OR = 0.178, 95% CI 0.042–0.749).

**Table 3:** Association between the CMA1 haplotypes and atrial fibrillation

Name	Haplotype	Case (freq)	Control (freq)	Odds Ratio (95%CI)	*p*
Hap1	CGCTA	23(0.097)	33(0.145)	0.633 (0.360–1.116)	0.112
Hap2	CGTTA	1(0.004)	5(0.020)	0.212 (0.024–1.840)	0.122
Hap3	TACCA	2(0.010)	4(0.017)	0.613 (0.123–3.057)	0.547
Hap4	TACTG	50(0.214)	49(0.214)	0.995 (0.638–1.550)	0.981
Hap5	TATTG	2(0.010)	12(0.051)	0.178 (0.042–0.749)	0.008
Hap6	TGCCA	54(0.229)	49(0.213)	1.092 (0.704–1.694)	0.695
Hap7	TGTCA	3(0.014)	3(0.013)	1.090 (0.228–5.203)	0.914
Hap8	TGTTG	95(0.404)	67(0.290)	1.668 (1.132–2.458)	0.009

*CI* Confidence interval, *OR* Odds ratio. The haplotype structure of the CMA1 gene was rs1956921 (C/T), rs1800875 (A/G), rs1800876 (T/C), rs5244 (C/T) and rs1885108 (A/G). Haplotypes with frequencies < 0.01 were not included in the table

## Discussion

SNPs present in > 1% of the population are common in the human genome, and have been shown to affect gene function and influence susceptibility to certain diseases. Numerous recent studies have demonstrated that AF is a genetically heterogeneous disease [25]. In the current study, we sequenced five SNPs of the *CMA1* gene in 126 unrelated Han Chinese patients diagnosed with lone AF. To the best of our knowledge, this represents the first study to demonstrate the possible contribution of *CMA1* polymorphisms to the pathogenesis of AF. We found a possible association between the rs1800875 GG genotype and an increased risk of AF. Haplotype analysis further demonstrated that the rs1800875-G haplotype (Hap 8 TGTTG) might lead to an increased risk of AF, while rs1800875-A (Hap 5 TATTG) was associated with a reduced risk of AF.

This case-control study demonstrated a significant difference in the frequencies of the *CMA1* rs1800875 GG genotype between AF patients and controls, indicating that this genotype might be a susceptibility factor for AF in the Chinese Han population, and further suggesting that the function of the *CMA1* gene may be modulated by this polymorphism. Orlowska-Baranowska et al. [26] found that the rs1800875 SNP was an independent predictor of left ventricular mass in male patients with aortic stenosis. Amir et al. reported [27] that rs1800875 was related to a non-ischemic etiology of heart failure, and that patients homozygous for the G allele had a significantly greater reduction in left ventricular systolic function. Both of these studies indicated an association between rs1800875 and cardiac remodeling. However, few studies have investigated the molecular pathogenesis and functional role of the rs1800875 G allele in cardiac remodeling. Growing evidence suggests that cardiac Ang II formation is predominantly dependent on chymase [9, 10].

The present study found no significant associations between the genotype and allele distributions of the rs1800876, rs1885108, rs1956921, and rs5244 SNPs and AF. Previous reports also suggested that some SNPs had no effect on gene function and phenotype [28]. However, further investigations with larger sample sizes are needed to confirm these findings.

The main limitation of the current study was the relatively small number of patients. Furthermore, functional studies are required to elucidate the details of the molecular mechanisms whereby the SNP or haplotype affects the *CMA1* gene. In addition, the present study only enrolled Chinese Han individuals from Hangzhou, and further studies are needed to determine if the results can be generalized to other racial groups and locations.

## Conclusion

In summary, the results of this study may help in the identification of polymorphisms related to AF in the Chinese Han population. Some *CMA1* polymorphisms appear to be associated with AF, and the rs1800875 GG genotype might serve as a susceptibility factor for AF. A better understanding of the genetic mechanisms of AF will allow more accurate risk stratification of AF patients. The recurrence rates of AF are approximately 40–50% after a single procedure and 10–20% after multiple procedures (including medication, electrical cardioversion, or catheter ablation) [29], and the current results might provide clues to aid the development of personalized therapeutic strategies in these patients. However, the exact mechanisms underlying this risk increment remain poorly elucidated, and further studies are needed to clarify the roles of the rs1800875, rs1800876, rs1885108, rs1956921, and rs5244 *CMA1* polymorphisms in AF.

## Data Availability

The dataset analyzed during the current study are available from the corresponding author on reasonable request.

## Abbreviations

AF: Atrial fibrillation
Ang II: Angiotensin II
BMI: Body mass index
CMA1: Chymase 1 gene
Glu: Glucose
LDL: Low density lipoprotein
OR: Odds ratio
PCR: Polymerase chain reaction
RAS: Renin-angiotensin system
SNPs: S ingle nucleotide polymorphisms
TC: Total cholesterol
TG: Triglyceride
